# Same-admission versus delayed cholecystectomy for mild acute biliary pancreatitis: a systematic review and meta-analysis

**DOI:** 10.1186/s12893-018-0445-9

**Published:** 2018-11-29

**Authors:** Yun-Xiao Lyu, Yun-Xiao Cheng, Hang-Fei Jin, Xin Jin, Bin Cheng, Dian Lu

**Affiliations:** Department of Hepatobiliary Surgery, Dongyang People’s Hospital, 60 West Wuning Road, 322100, Dongyang, Zhejiang, China

**Keywords:** Pancreatitis, Cholecystectomy, Laparoscopic, Review, Meta-analysis

## Abstract

**Background:**

The timing of laparoscopic cholecystectomy (LC) performed after the mild acute biliary pancreatitis (MABP) is still controversial. We conducted a review to compare same-admission laparoscopic cholecystectomy (SA-LC) and delayed laparoscopic cholecystectomy (DLC) after mild acute biliary pancreatitis (MABP).

**Methods:**

We systematically searched several databases (PubMed, EMBASE, Web of Science, and the Cochrane Library) for relevant trials published from 1 January 1992 to 1 June 2018. Human prospective or retrospective studies that compared SA-LC and DLC after MABP were included. The measured outcomes were the rate of conversion to open cholecystectomy (COC), rate of postoperative complications, rate of biliary-related complications, operative time (OT), and length of stay (LOS). The meta-analysis was performed using Review Manager 5.3 software (The Cochrane Collaboration, Oxford, United Kingdom).

**Results:**

This meta-analysis involved 1833 patients from 4 randomized controlled trials and 7 retrospective studies. No significant differences were found in the rate of COC (risk ratio [RR] = 1.24; 95% confidence interval [CI], 0.78–1.97; *p* = 0.36), rate of postoperative complications (RR = 1.06; 95% CI, 0.67–1.69; *p* = 0.80), rate of biliary-related complications (RR = 1.28; 95% CI, 0.42–3.86; *p* = 0.66), or OT (RR = 1.57; 95% CI, − 1.58–4.72; *p* = 0.33) between the SA-LC and DLC groups. The LOS was significantly longer in the DLC group (RR = − 2.08; 95% CI, − 3.17 to − 0.99; *p* = 0.0002). Unexpectedly, the subgroup analysis showed no significant difference in LOS according to the Atlanta classification (RR = − 0.40; 95% CI, − 0.80–0.01; *p* = 0.05). The gallstone-related complications during the waiting time in the DLC group included gall colic, recurrent pancreatitis, acute cholecystitis, jaundice, and acute cholangitis (total, 25.39%).

**Conclusion:**

This study confirms the safety of SA-LC, which could shorten the LOS. However, the study findings have a number of important implications for future practice.

## Background

Acute pancreatitis is a common disease in the emergency room with an annual incidence ranging from 4.9 to 35 per 100,000 population [1]. According to the Atlanta classification, 80% of patients with pancreatitis have mild acute pancreatitis [2]. Acute biliary pancreatitis is one of the most common types of acute pancreatitis, accounting for up to 40 to 70% of cases [3–5].

Cholecystectomy is considered to be effective in reducing the recurrence of acute gallstone pancreatitis [6–8]. Previous studies have shown that the probability of recurrence of acute pancreatitis without cholecystectomy is as high as 33% [9]. However, a primary concern in the treatment of mild acute biliary pancreatitis (MABP) is the optimal timing of LC. A previous review showed that delayed cholecystectomy can increase readmission [10–12]. However, most guidelines advise early LC after MABP [12–14]. Early LC can reduce the risk of recurrent biliary events [15]. However, many previous studies had low methodological quality. One of the heterogeneities is the previous study using different criteria for the severity of pancreatitis. We defined same-admission LC (SA-LC) as LC performed within the same admission after MABP. A nationwide randomized study was recently published [16]. The aim of this study was to compare SA-LC and DLC after MABP through analysis including recently studies.

## Methods

### Search strategy

Two authors independently performed a systematic review of PubMed, Embase, Web of Science, and the Cochrane Library from 1 January 1992 to 1 June 2018. The search terms were “cholecystectomy,” “pancreatitis,” “laparoscopy,” and “laparoscopic cholecystectomy.” In this meta-analysis, we defined SA-LC as initial LC performed during the same admission because of MABP. The control group underwent DLC at readmission. The references of the articles identified after the initial search were also manually reviewed. The language in the search was limited to English. This meta-analysis adhered to the PRISMA statement [17].

### Inclusion and exclusion criteria

We applied the following inclusion criteria: (1) trials comparing the clinical outcomes of interest between SA-LC and DLC, (2) studies with a clear MABP severity scoring system, and (3) studies that provided adequate data on the clinical outcomes.

We excluded studies that (1) were review articles, case reports, abstracts, editorials, and letters to the editor; (2) included patients with severe pancreatitis and pancreatitis of other origins.

### Outcomes of interest

The outcome measures were the rate of conversion to open cholecystectomy (COC), operative time (OT), length of stay (LOS), rate of postoperative complications, and rate of biliary-related complications. Biliary-related complications were common bile duct injury and bile leakage of any cause. Gallstone-related events were defined as complications that occurred during the waiting time.

### Data extraction

Two investigators extracted the following original data from the literature onto a standardized form: the authors, year of publication, type of study, country, definitions of SA-LC and DLC, criteria of pancreatitis, and outcomes of interest. If necessary, the author or authors of the study were contacted to obtain the study data. Conflicts in data abstraction were resolved by consensus and reference to the original article.

### Quality assessment

The Newcastle–Ottawa Scale was used to evaluate the methodological quality of the included trials [18]. The scale ranges from 0 to 9 points; studies with a score of ≥6 are considered to have high methodological quality.

### Statistical analysis

All statistical analyses were performed using Review Manager (RevMan) version 5.3 software (The Cochrane Collaboration, Oxford, United Kingdom). The risk ratio (RR) and 95% confidence interval (CI) were used to describe dichotomous outcomes. The median and range or interquartile range were used to estimate the mean and standard deviation using a formula from a previous study [19]. The I^2^ index was used as an indicator of between-study heterogeneity. A fixed-effects model was used when I^2^ < 50%; otherwise, a random-effects model was used. A two-tailed *p* value of < 0.05 was considered statistically significant.

## Results

### Selected studies and characteristics of the trials

**Fig. 1:**
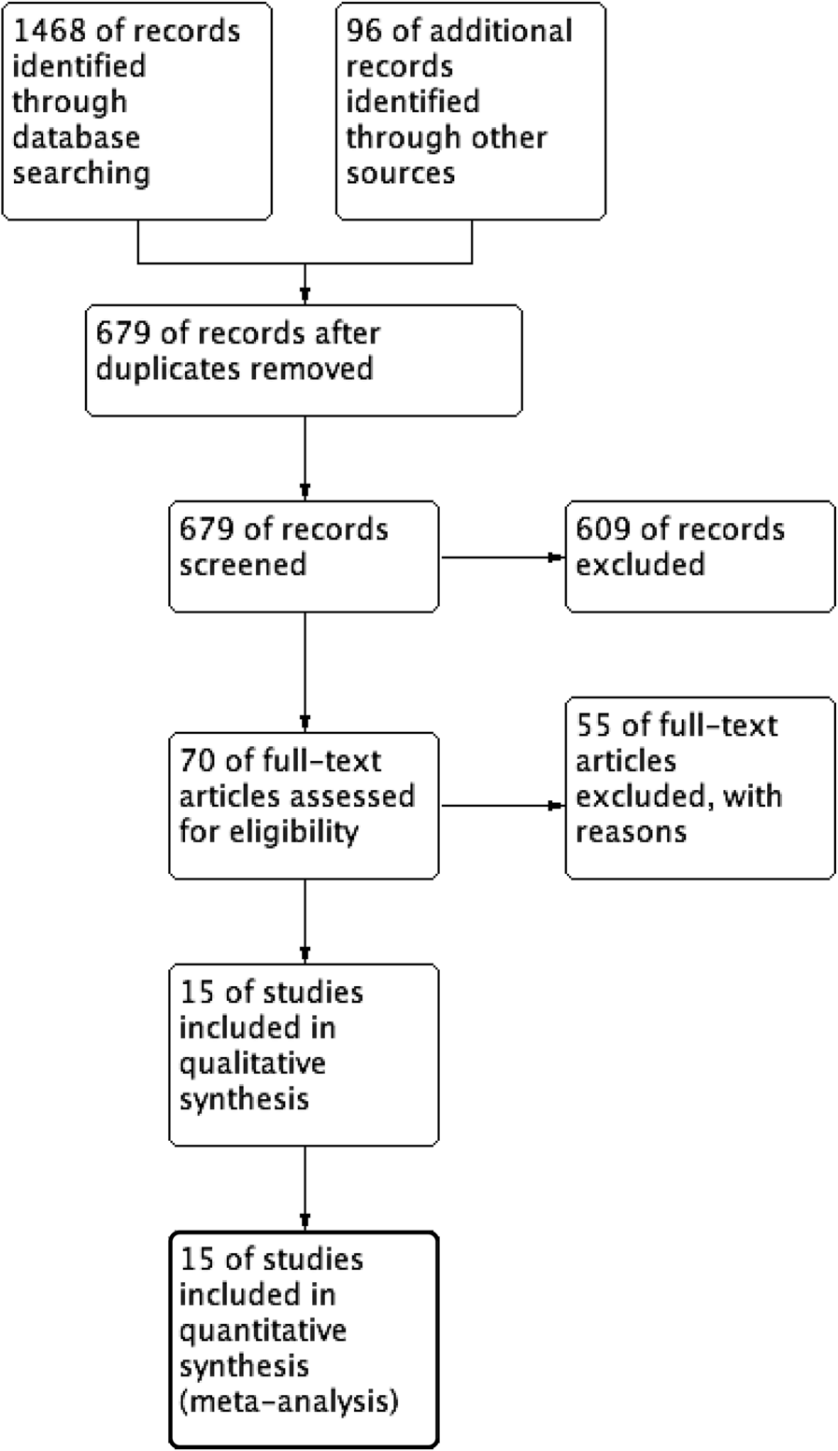
Flow diagram of the published articles evaluated for inclusion in this meta-analysis

A flow chart of this study is shown in Fig. 1. The search strategy yielded 1564 papers from the respective search sources, of which 679 duplicate references were excluded. The remaining 885 studies were retrieved to examine their titles and abstracts, resulting in 15 articles that appeared to meet our selection criteria. Of these articles, four were excluded because one study did not provide the criteria of pancreatitis [20] and three studies included patients with severe pancreatitis [21–23]. Finally, 11 trials [16, 20, 24–33] (4 randomized controlled trials [RCTs] [16, 24, 30, 32] and 7 retrospective studies [20, 25–29, 31, 33]) involving 1833 participants were included in the meta-analysis.

The 1833 patients were divided into either the SA-LC group (*n* = 913) or DLC group (*n* = 920). The sample sizes ranged from 44 to 316. The countries involved were the United States, Turkey, the United Kingdom, the Netherlands, Italy, Switzerland, Sweden, India, Malaysia, and Canada. The Ranson score and Atlanta classification severity criteria of MABP were applied in the included studies. The main characteristics of the studies included in this meta-analysis are presented in Table 1.

**Table 1:** Characteritics of included studies

Author	Country	Study design	Sample		Definition		Criteria of MABP	NOS
			SA-LC	DLC	SA-LC	DLC		
Aboulian et al. 2010 [24]	USA	RCT	25	25	< 48 h	> 48 h	Ranson score	8
Aksoy et al. 2017 [25]	Turkey	Retrospective	75	87	< 3 days	4–10 weeks	Ranson scor	8
Al-Qahtani et al. 2014	Saudi Arabia	Retrospective	267	83	Index admission	6–12 weeks	Ranson score	7
Costa et al. 2015 [16]	Dutch	RCT	128	136	> 3 days	25–30 days	Atlanta classification	8
Falor et al. 2012 [27]	USA	Retrospective	117	186	< 48 h	> 48 h	Ranson score	7
Griniatsos et al. 2005 [28]	UK	Retrospective	20	24	< 2 weeks	> 2 weeks	Ranson score^a^	7
Guadagni et al. 2017 [29]	Italy	Retrospective	98	218	< 3 days	> 3 days	Ranson score	8
Jee et al. 2016	Malaysia	RCT	38	34	Same admission	> 6 weeks	Atlanta classification	8
Nebiker et al. 2009 [31]	Switzerland	Retrospective	32	67	< 14 days	> 14 days	Ranson score	8
Rozh Noel et al. 2018	Sweden	RCT	32	34	Index admission	> 6 weeks	Atlanta classification	8
Sinha et al. 2008 [33]	India	Retrospective	81	26	< 7 days	> 6 weeks	Ranson’s score	7

*RCT* randomized controlled trial, *SA-LC* same-admission laparoscopic cholecystectomy, *DLC* delayed laparoscopic cholecystectomy

^a^Modified Glasgow Scoring System

### Outcomes

#### COC

**Fig. 2:**
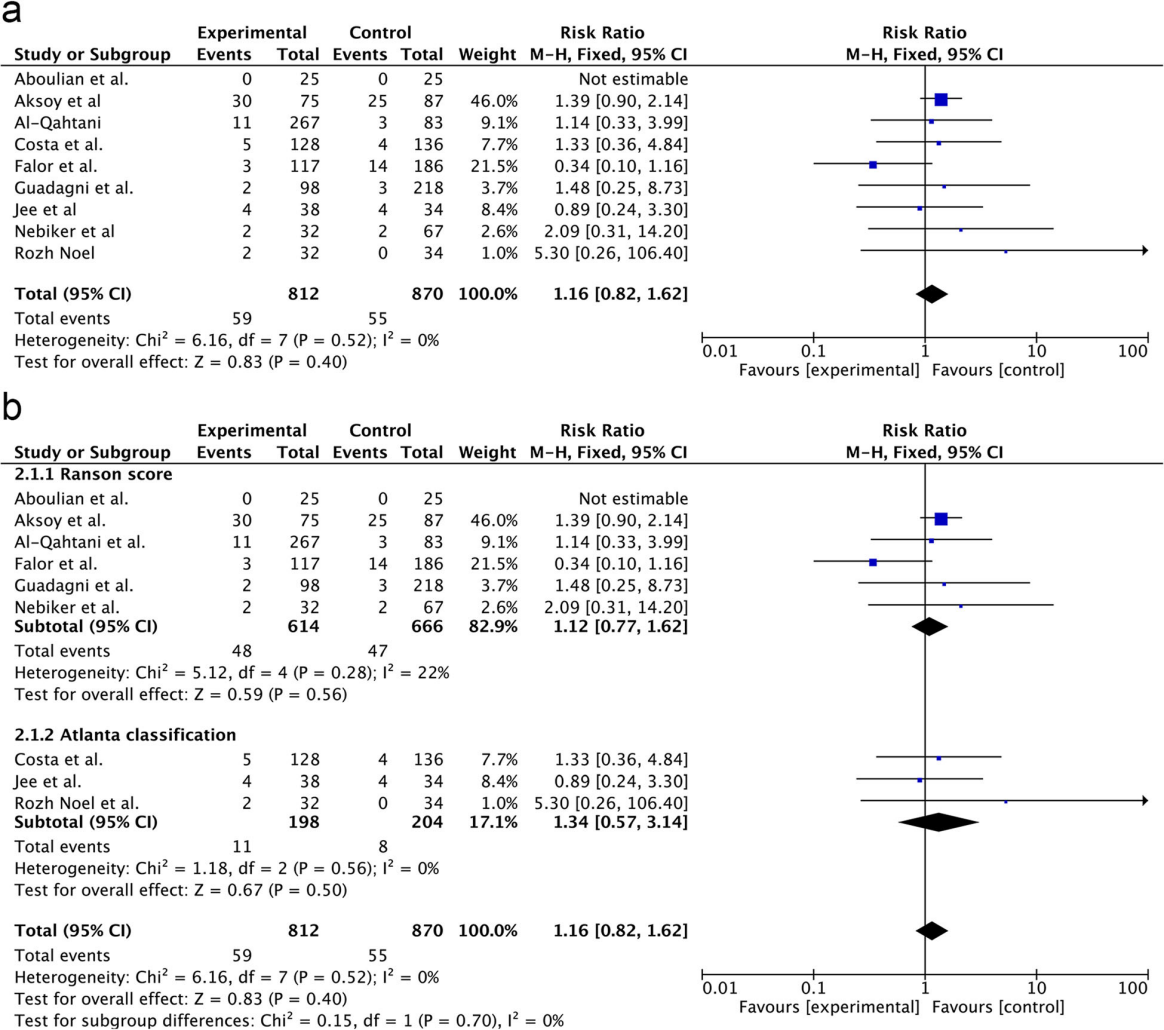
Forest plot of the meta-analysis comparing SA-LC and DLC regarding the incidence of COC (**a**. all;**b**. subgroup of MABP criteria).

Data regarding COC were provided in nine studies. The rate of COC was 7.27% (59/812) in the SA-LC group and 6.32% in the DLC group (55/870). There was no significant difference between the two groups (RR = 1.24; 95% CI, 0.78–1.97; *p* = 0.36) (Fig. 1a). According to the different criteria of MABP, the subgroup analysis showed no significant differences between the SA-LC group (RR = 1.12; 95% CI, 0.77–1.62; *p* = 0.56) and DLC group (RR = 1.34; 95% CI, 0.57–3.14; *p* = 0.50) in the two subgroups (*p* = 0.70) (Fig. 2b).

#### Postoperative complications

**Fig. 3:**
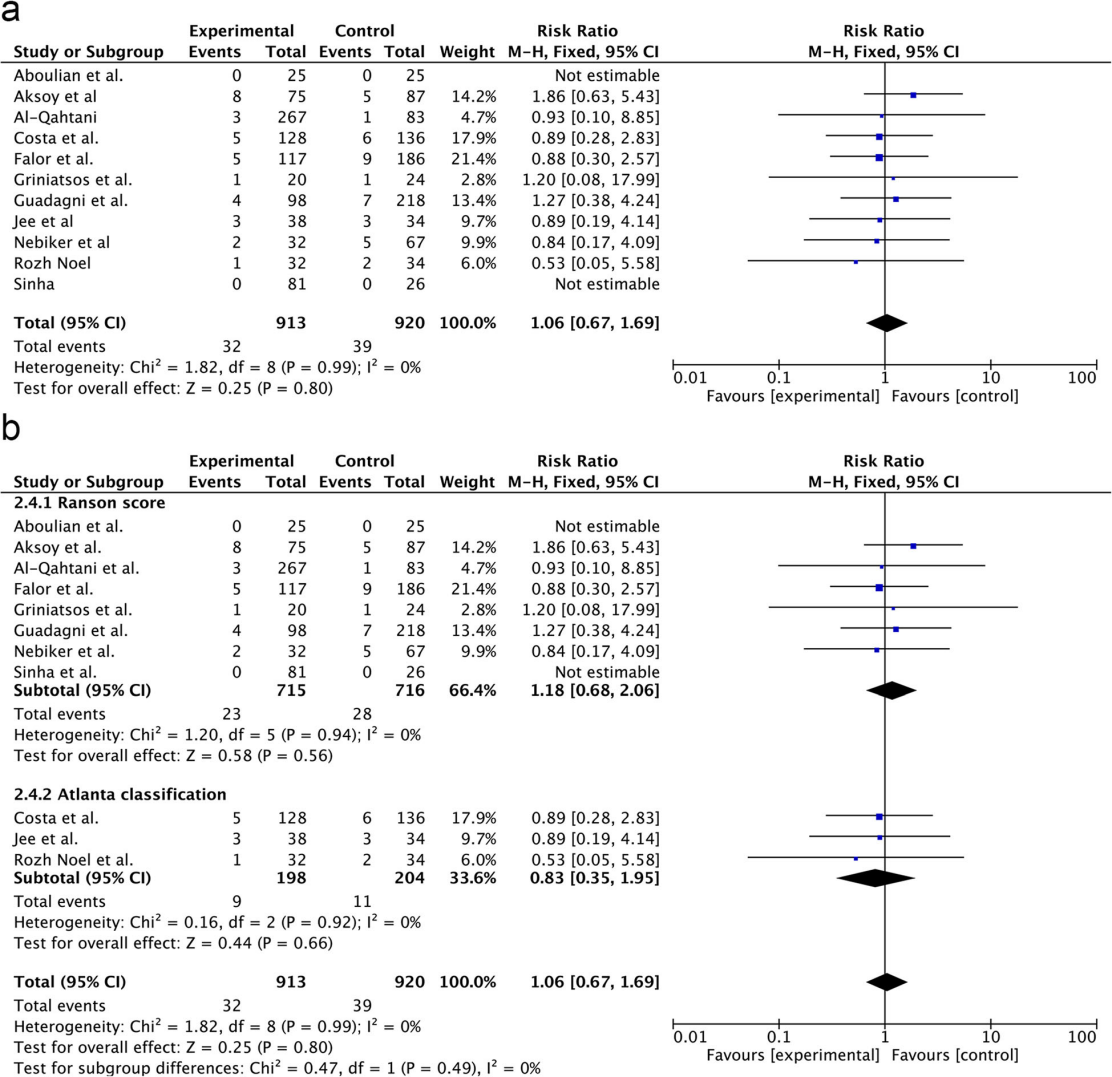
Forest plot of the meta-analysis comparing SA-LC and DLC regarding the incidence of postoperative complication (**a**. all; **b**. subgroup of MABP criteria).

All 11 studies provided complete data on postoperative complications. The meta-analysis showed no significant differences in the incidence of postoperative complications between the SA-LC and DLC groups (RR = 1.06; 95% CI, 0.67–1.69; *p* = 0.80) (Fig. 3a). Similar results were obtained in the subgroup analysis (RR = 1.18; 95% CI, 0.68–2.06; p = 0.56 vs. RR = 0.83; 95% CI, 0.35–1.95; *p* = 0.66) (Fig. 3b).

#### Biliary-related complications

**Fig. 4:**
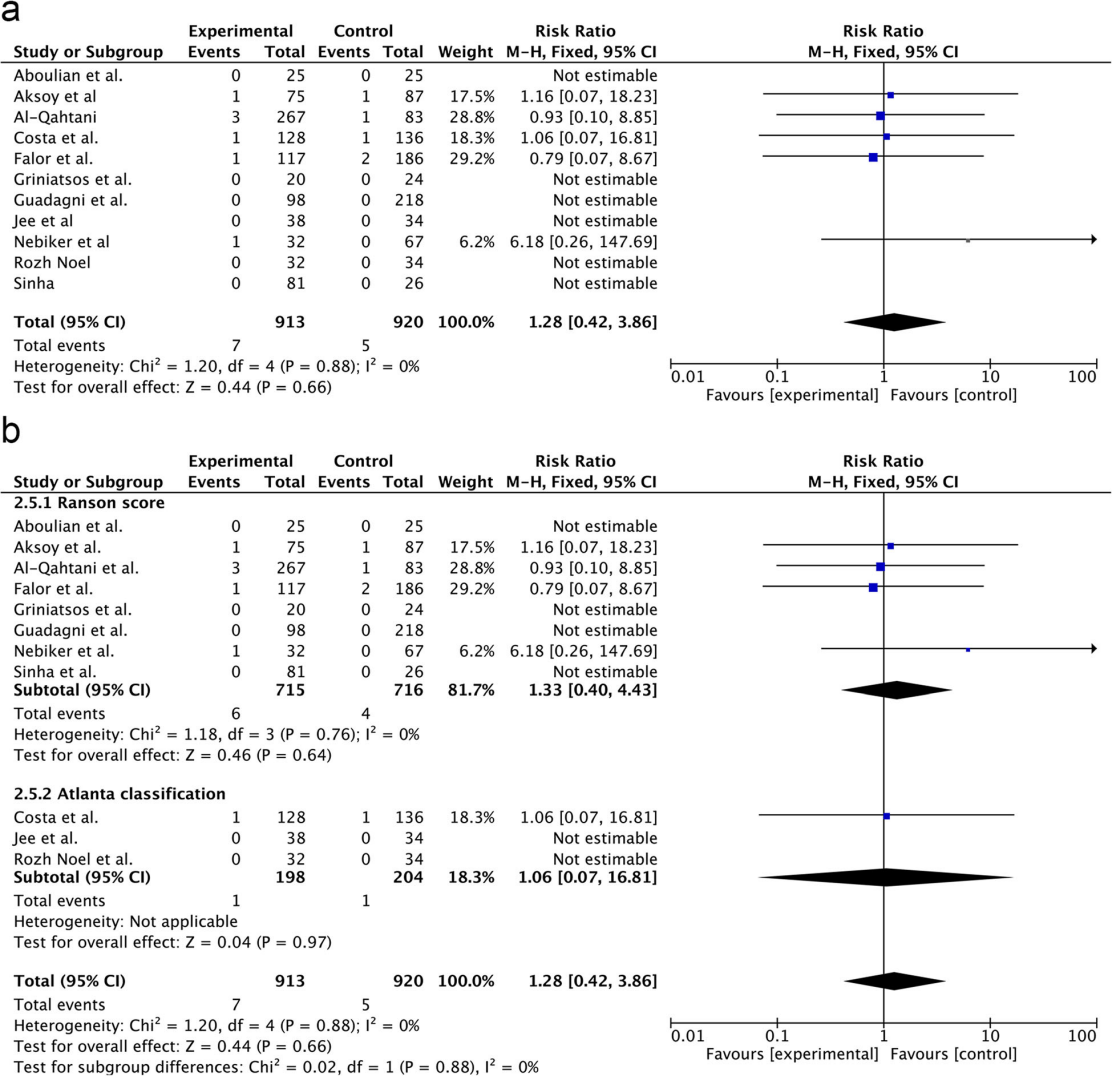
Forest plot of the subgroup meta-analysis of the incidence of biliary-related complication (**a**. all;**b**. subgroup of MABP criteria).

The incidence of biliary-related complications was 7/913 and 5/920 in the SA-LC and DLC group, respectively (RR = 1.28; 95% CI, 0.42–3.86; p = 0.66) (Fig. 4a). The result of the Ranson score subgroup analysis showed no significant differences between the SA-LC and DLC groups (RR = 1.33; 95% CI, 0.40–4.43; *p* = 0.64) (Fig. 4b). Similarly, the incidence of biliary-related complications in the Atlanta classification subgroup analysis was not significantly different between the two groups (RR = 1.06; 95% CI, 0.07–16.81; *p* = 0.97) (Fig. 4b).

#### OT

**Fig. 5:**
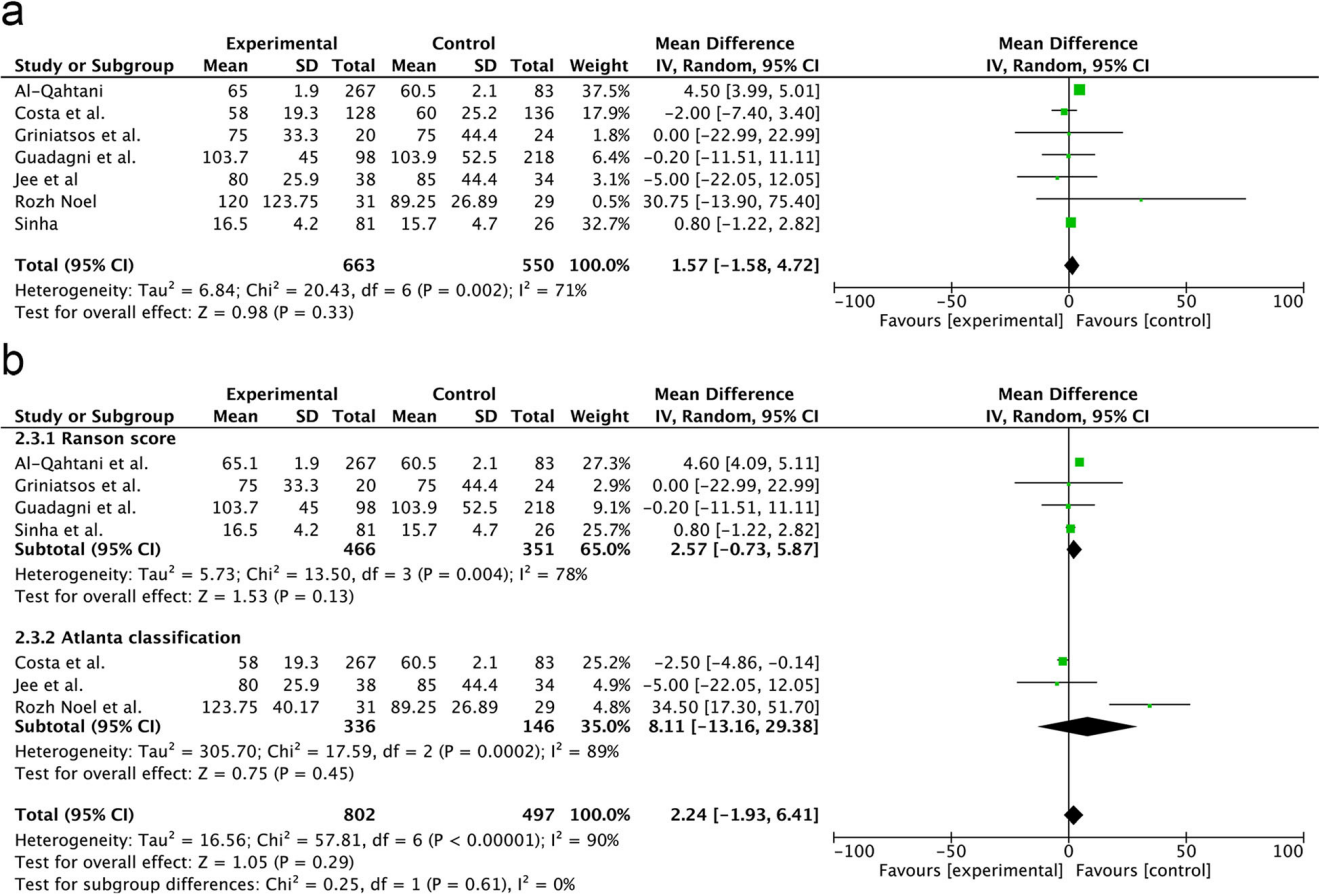
Forest plot of the meta-analysis comparing SA-LC and DLC regarding the incidence of operative time (**a**. all;**b**. subgroup of MABP criteria).

The OT was not significantly different between the groups (RR = 1.57; 95% CI, − 1.58–4.72; *p* = 0.33) (Fig. 5a). There was no difference in the OT in the Ranson score subgroup analysis (RR = 2.57; 95% CI, − 0.73–5.87; *p* = 0.13) and Atlanta classification (RR = 8.11; 95% CI, − 13.16–29.38; *p* = 0.45) (Fig. 5b).

#### Los

**Fig. 6:**
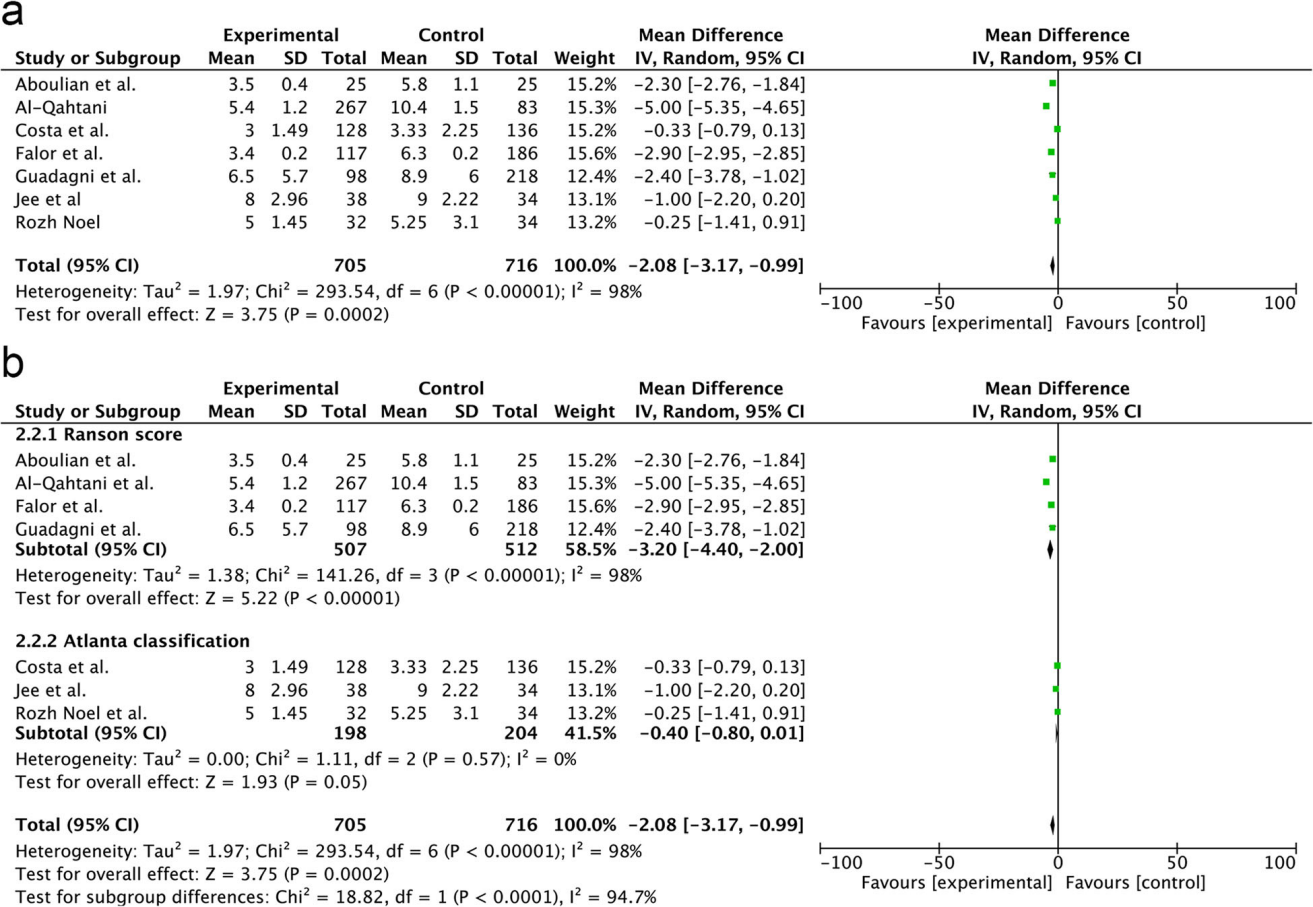
Forest plot of the meta-analysis comparing SA-LC and DLC regarding the incidence of length of hospital stay (**a**. all;**b**. subgroup of MABP criteria)

Seven trials provided data regarding LOS. Our study showed that SA-LC could significantly shorten the LOS (RR = − 2.08; 95% CI, − 3.17 to − 0.99; *p* = 0.0002) (Fig. 6a). In the Ranson subgroup analysis, the LOS in the SA-LC group was shorter than that in the DLC group (RR = − 3.20; 95% CI, − 4.40 to − 2.00; *p* < 0.00001) (Fig. 6b). In the Atlanta subgroup analysis, the LOS in the SA-LC group was not significantly different from that in the DLC group (RR = − 0.40; 95% CI, − 0.80–0.01; *p* = 0.05). There was a significant difference between the two subgroups (p = 0.0002) (Fig. 6b).

#### Gallstone-related events

An analysis of the full text of all included studies showed that in the DLC group, the most common gallstone-related event during the waiting time was biliary colic, which occurred in approximately 13.56% of patients (86/634). Other events were recurrent acute pancreatitis (54/634), acute cholecystitis (10/634), jaundice (7/634), and acute cholangitis (4/634). In the included literature, the probability of stone-related events during the waiting period was about 25.39% (Table 2).

**Table 2:** Gallstone-related complications during the waiting time

Author (sample of DLC)	AP	BC	AC	Jaundice	Cholangitis	Total
Al-Qahtani et al (*n* = 267)	9		2	7	2	20
Costa et al (*n* = 128)	12	62	2		2	78
Falor et al. (*n* = 117)	1					1
Griniatsos et al. (*n* = 20)	1	6				7
Jee et al (*n* = 38)	2	10	3			15
Nebiker et al (*n* = 32)	9	4	2			15
Rozh Noel et al (*n* = 32)	5	4				9
Total (*n* = 634)	54	86	10	7	4	161

*SA-LC* same-admission laparoscopic cholecystectomy, *AP* acute pancreatitis, *BC* biliary colic, *AC* acute cholecystitis

## Discussion

The current study comparing SA-LC with DLC showed the mean rate of COC, rate of postoperative complications, rate of biliary-related complications, and OT. The LOS in the SA-LC group was shorter than that in the DLC group. Given some limitations of the present analysis, future studies on the current topic are recommended.

Several multifactorial scoring systems have been used to classify the severity of acute pancreatitis in the previous studies. Each of these scoring systems has its own limitations, including low sensitivity and specificity [34]. To our knowledge, this is the first analysis of postoperative complications, OT, and LOS according to different grading criteria. Interestingly, in the Atlanta subgroup analysis, there was no significant difference in hospitalization time between the SA-LC and DLC groups. This difference may be related to the sensitivity and specificity of the different scoring systems. However,in the other outcomes, there were no significantly differences between two subgroups. Future studies should further clarify the impact of these different scoring systems.

LC has become the gold standard surgical approach for the treatment of gallbladder disease [35].The possible increase in the COC rate is considered to be the reason why many surgeons choose DLC [36, 37]. A previous study demonstrated that early LC may be more technically challenging because of the edema [33]. In our study, we found that the rate of COC was 7.3% in the SA-LC group which similar to previous studies. In a study by Aksoy et al., the main reason for COC in the early group was obscure anatomy (including Calot’s triangle), and no significant differences from the delayed group were observed [25]. Interestingly, a study by Sinha showed that dissection of Calot’s triangle is more difficult in DLC [33].

However, a certain proportion of complications may still appear after LC, especially in the acute phase [38, 39]. Some researchers believe that LC during the same admission increases the severity of edema caused by pancreatitis [10]. A multicenter study showed that LC within 2 weeks of acute biliary pancreatitis could increase postoperative complications (3% vs. 1%) [36]. In contrast, the current study showed that SA-LC did not increase postoperative complications. A recent review concluded that the rate of postoperative complications in the early LC group was lower than that in the DLC group [40]. One of the most important types of postoperative complications after LC are biliary-related complications, which have a negative impact on patient survival and quality of life [41, 42]. Prior studies have reported rates of biliary-related complications ranging from 0.2 to 1.5% after LC [43, 44]. The results of a cohort study showed that the incidence of major complications associated with SA-LC, including common bile duct injury and bile leakage, was twice that associated with DLC [36]. However, the sample of patients with major complications was small in this previous study. In the present study, the analysis of biliary-related complications showed no significant differences. Unlike these previous studies, our meta-analysis excluded trials involving patients with severe pancreatitis. LC performed in patients with severe pancreatitis may be unsafe [45].

Surgical time is an indicator of the degree of difficulty in surgery. Similar to previous studies, the present study showed that the timing of LC did not affect the surgical time. Only a few studies provided complete data regarding the mean and standard deviation of the LOS, and we estimated the data using a formula. Therefore, the conclusions regarding the OT and hospital stay still need to be interpreted with caution.

High readmission rates were found in previous studies, ranging from 15 to 29% [8, 10, 46–49]. Gallstone-related complications included acute cholecystitis, recurrent pancreatitis, and biliary colic. Although biliary colic is the most common complication during the waiting period, recurrent pancreatitis remains the most serious event and reason for readmission [6]. In an up-to-date large-scale RCT, the rates of readmission for gallstone-related complications was 12% in the DLC group [16]. A nationwide analysis showed that readmission for acute pancreatitis is most often due to recurrent acute pancreatitis [6]. In the present study, the probability of recurrent pancreatitis reached 8.5%. Our analysis of these differences may be related to the fact that in a retrospective study, surgeons are able to perform LC during hospitalization in patients who are susceptible to recurrent pancreatitis.

Limitations: First, despite the fact that LC was performed during the same admission, the specific OT was different. Second, of the included studies, only four were RCTs; the rest were retrospective studies, and heterogeneity was present among these retrospective studies. Third, some of the research data were obtained using a formula. A large-scale RCT is currently in progress [50].

## Conclusions

In summary, SA-LC for MABP can reduce the hospital LOS and does not increase the incidence of postoperative complications. However, this conclusion needs to be verified by higher-quality research.

## Abbreviations

COC: Conversion to open cholecystectomy
DLC: Delayed laparoscopic cholecystectomy
LC: Laparoscopic cholecystectomy
LOS: Length of stay
MABP: Mild acute biliary pancreatitis
OT: Operative time
RR: Risk ratio
SA-LC: Same-admission laparoscopic cholecystectomy
